# Can molecular mimicry explain the cytokine storm of SARS‐CoV‐2?: An in silico approach

**DOI:** 10.1002/jmv.27040

**Published:** 2021-06-11

**Authors:** Gustavo Obando‐Pereda

**Affiliations:** ^1^ Proteomics, Inflammation and Pain Laboratory Universidad Católica de Santa María Arequipa Peru

**Keywords:** cytokine storm, macrophage, molecular mimicry, SARS‐CoV‐2

## Abstract

PARP14 and PARP9 play a key role in macrophage immune regulation. SARS‐CoV‐2 is an emerging viral disease that triggers hyper‐inflammation known as a cytokine storm. In this study, using in silico tools, we hypothesize about the immunological phenomena of molecular mimicry between SARS‐CoV‐2 Nsp3 and the human PARP14 and PARP9. The results showed an epitope of SARS‐CoV‐2 Nsp3 protein that contains consensus sequences for both human PARP14 and PARP9 that are antigens for MHC Classes 1 and 2, which can potentially induce an immune response against human PARP14 and PARP9; while its depletion causes a hyper‐inflammatory state in SARS‐CoV‐2 patients.

## Highlights

- Molecular mimicry could produce an inflammatory state in SARS‐CoV‐2 patients.
- Human PARP14 and PARP9 are the proteins involves in this phenomena.

## 1 INTRODUCTION

SARS‐COV‐2 was described in December 2019 as being the result of zoonotic transmission from wild animals traded in the Wuhan market^1^ to humans presenting primarily symptoms such as fever, nonproductive cough, and myalgia or fatigue, normal or decreased leukocyte counts, and radiographic evidence of pneumonia.^2^ On January 7, 2020, the World Health Organization named this virus New Coronavirus 2019 (2019‐nCoV). However, the genome of this virus has an 86.9% similarity with the Severe Acute Respiratory Syndrome – CoV (SARS‐CoV) genome,^3^ and researchers changed the initial name to Severe Acute Respiratory Syndrome Corona Virus‐2 (SARS‐CoV‐2).^4^ In a year, by March 21, 2021,^5^ this new disease produced a pandemic with 122.524 million cases and over 2.7 million deaths worldwide.

The SARS‐CoV‐2 is an enveloped positive‐sense RNA virus belonging to the *Coronaviridae*, genus *Betacoronavirus* 6, 7 family. The SARS‐CoV‐2 genome contains 14 open reading frames (ORFs), 4 encoded structural proteins, spikes (S), a membrane (M), an envelope (E), and a nucleocapsid (N) that constitute a protective shell surrounding the genetic material.^8^ Other remaining proteins, such as the NsP1‐16 and 9 other accessory proteins enhance its virulence.^8^ The nonstructural protein 3 (Nsp3) is an important protein for the virus to process viral polyproteins and build a fully functional complex allowing viral propagation.^9^ Another function of the Nsp3 is its significant role in regulating the host's inflammatory and immune response.^1^ Several studies demonstrate that the Nsp3 and the human poly‐adenosine diphosphate‐ribose polymerase 9 (parp9)^10^ and parp14^1^ have identical residues that could produce molecular mimicry, leading to leukopenia and an altered inflammatory response.^1^, ^10^ This condition can be explained by a cytokine storm state, related to the macrophage activation syndrome (MAS).^11^, ^12^

The PARPs are a family of important enzymes that catalyze posttranslational ribosylation modification of proteins using NAD^+^ as a substrate to carry out mono or poly ADP‐ribosylation modification on target proteins to trigger many processes of cellular metabolism, such as DNA repair,^13^ regulation of disease pathogenesis,^14^ modulation of immune response^1^ and is involved in viral infections.^15^ Seventeen PARP family members have been described,^16^ the most important in immune regulation being PARP14 and PARP9.^13^, ^17^ PARP14 increases IL‐4 induced cytokine through STAT6,^14^ responsible for anti‐inflammatory macrophage activation (M2),^17^ immune homeostasis,^1^, ^13^ tissue injury and inflammatory macrophage regeneration and regulation (M1).^17^ PARP9 as PARP14 is responsible for macrophage activation.^17^ In the SARS‐COV‐2 disease, a molecular mimicry phenomenon has been observed, and two recent studies have reported this phenomenon between viral proteins against PARP9^10^ and PARP14.^18^ The aim of this study is to analyze the molecular mimicry phenomena using in silico tools between SARS‐Cov‐2 and human proteins.

## 2 METHODS

### 2.1 Basic local alignment search tool

The selected Sars‐Cov‐2 protein sequences: Nsp2 (YP_009742609.1), Nsp3(YP_009742610.1), Nsp4 (YP_009742611.1), Nsp6 (YP_009742613.1), Nsp7 (YP_009742614.1), Nsp8 (YP_009742615.1), Nsp9 (YP_009742616.1), Nsp10 (YP_009742617.1), Nsp11 (YP_009725312.1), Orf1ab (YP_009724389.1), Orf1a (YP_009725295.1), Orf3 (YP_009724391.1), Orf6 (YP_009724394.1), Orf7a (YP_009724395.1), Orf7b (YP_009725318.1), Orf8 (YP_009724396.1), Orf10 (YP_009725255.1), S (YP_009724390.1), E (YP_009724392.1), M (YP_009724393.1), and N (YP_009724397.2); were submitted to the BLASTp tool, and a sequence similarity search was performed in the human proteome database. Subsequently, a second individual sequence analysis was performed using the DNASTAR Lasergene software for similar proteins.^19^ Sequence alignment was performed using the Clustal Omega server with default parameters.^20^

### 2.2 Prediction of B cell, cytotoxic T lymphocytes, and helper T lymphocytes epitopes

The B‐cell epitopes were predicted using the BepiPred server and the Elliprot server to examine the epitope position in a 3D structure.^21^ Cytotoxic T lymphocyte (CTL) epitopes were predicted using the IEDB MHC I algorithm (http://tools.iedb.org/mhci) and helper T lymphocyte (HTL) epitopes were predicted using the MHC II binding prediction tools (http://tools.iedb.org/mhcii). The antigenic properties of the epitopes were studied using the Vaxijen 2.0 server at a threshold of 0.4. Peptide toxicity was predicted from the ToxinPred server (http://crdd.osdd.net/raghava/toxinpred/), and allergenicity was predicted from the AllegernFP 1.0 server (http://ddg-pharmfac.net/AllergenFP/). All of these analyses were taken into account to select the epitopes.

### 2.3 Hydrophobic and antigenic protein analysis

The hydrophobic and antigenic analysis was performed using the Kyte–Doolittle and Jameson–Wolf algorithms of human and viral protein. To determine whether the epitope found was located on the outer surface of the protein where antigen–antibody formation occurs, an overlay of the predicted epitopes and the results of hydrophobic and antigenic analysis was performed.^22^ The DNASTAR Protean program was used for this method.

### 2.4 Protein modeling and molecular docking

The three‐dimensional modeling of PARP9 was performed using the I‐TASSER online server (https://zhanglab.ccmb.med.umich.edu/I-TASSER/), while the *Z*‐score was used to verify the quality of the 3D protein modeling to select the best model.^23^ Human PARP14 and Sars‐Cov‐2 Nsp3 were extracted from PDB (3Q6Z and 6WEY, respectively).

In addition, the 3D structure of the selected epitopes was modeled with the PEPFOLD 3 server.^24^ Molecular docking of the peptides was performed using the DOCKTOPE server (http://tools.iedb.org/docktope/) for the HLA‐A*02:01 allele^25^ of MHC Class I and the CABS‐dock server (http://212.87.3.12/CABSdock/) for the HLA‐DR52c allele of MHC Class II (PBD: 3C5J).^26^ The HawKRank server was used for scoring.^27^

## 3 RESULTS

### 3.1 Basic local alignment search tool

**Figure 1:**
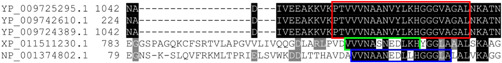
Basic local alignment showed epitopes of SARS‐CoV‐2 ORF1a, Nsp3, and ORF1ab with high similarity (in red). Human Parp14 (XP_011511230.1) and Parp9 (NP_001374802.1) showed a 14 aa with great similarity with SARS‐CoV‐2 ORF1a, Nsp3, and ORF1ab epitopes. Parp14 epitope framed in green and Parp9 epitope framed in blue were selected with prediction of B cell, cytotoxic T lymphocytes, and helper T lymphocytes epitopes tools

Protein basic local alignment search tool (BLAST) analysis showed the presence of a consensus sequence of fourteen amino acids that can be found in the Nsp3 protein, the ORF1a and ORF1ab of SARS‐CoV‐2 and in human Parp14 and Parp9, corresponding to amino acid positions Nsp3 (236–260), ORF1ab, and ORF 1a (1054–1078), 818–840 and 113–134 for human PARP14 and PARP9 respectively (Table 1 and Figure 1).

**Table 1:** Preserved epitopes between SARS‐CoV‐2 Nsp3 and human Parp9 and Parp14

PUBMED ref	Protein	Epitope	Length
XP_011511230.1	*Human PARP14 isoform X1*	VVVNA‐N—LKH‐GG‐A‐AL‐KA	818–840
NP_001374802.1	*Human PARP9 isoform d*	VVNAAN‐‐L‐HGGG‐A‐AL‐KA	113–134
YP_009725295.1	*ORF1a SARS‐CoV‐2*	PTVVVNAANVYLKHGGGVAGALNKA	1054–1074
YP_009742610.1	*Nsp3 SARS‐CoV‐2*		236–260
YP_009724389.1	*ORF1ab SARS‐CoV‐2*		1054–1074

### 3.2 Prediction of B cell, cytotoxic T lymphocytes, and helper T lymphocytes epitopes

**Figure 2:**
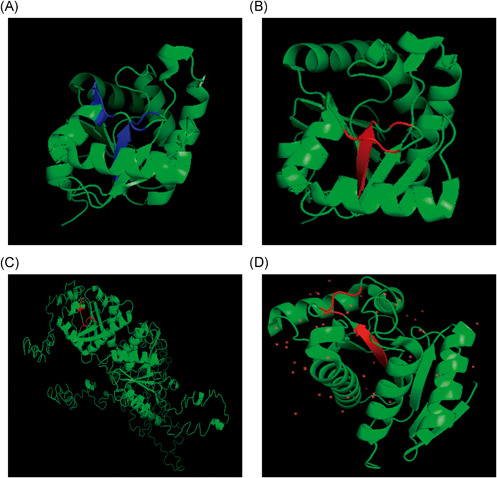
SARS‐CoV‐2 Nsp3 protein (PDB 6WEY) with consensus sequences with Parp9 in blue (A) and Parp14 in red (B). Human Parp9 (C) and human Parp14 (D) with consensus sequences in red. All epitopes are exposed

The predicted epitope PTVVVNAANVYLKHGGGVAGAL of Nsp3 (236–260), ORF1ab, and ORF1a (1054–1074) of SARS‐CoV‐2 containing the consensus amino acid sequence for human Parp14 (residues among 818–840) and Parp9 (residues among 113–134), showed enhanced activation of B‐cells by their allergenic nature (Figure 2). BepiPred showed that the epitopes have a large gradient and that the surface of the structure is exposed (Table 2). As mentioned, the predicted epitope of SARS‐CoV‐2, for CTL and HTL cells shows predicted epitopes with a high antigenic and allergenic property capable of inducing a large autoimmune response once recognized by the MHC Class I and MHC Class II allele numbers (Table 2).

**Table 2:** MHC‐I and MHC‐II alleles prediction epitopes

S/N	Core peptide	Length	MHCI alleles	MHCII alleles	Score/percentile	Antigenicity	Toxicity	Allergenicity
1	VVVNAANVYLKH	12	HLA‐B*15:01		0.752031	0.7077	Nontoxic	Allergen
			HLA‐A*30:02		0.529167			
			HLA‐B*35:01		0.5167			
2	VVNAANVYLKHGGGVA	16	HLA‐A*11:01		0.680557	0.6447	Nontoxic	Allergen
			HLA‐A*03:01		0.544704			
3	PTVVVNAANVYLKHGGGVAGA	21		HLA‐DRB1*13:02	**0.33**	0.5285	Nontoxic	Allergen
				HLA‐DRB1*13:02	**0.59**			

### 3.3 Hydrophobic and antigenicity prediction

Hydrophobic and antigenic analyses of SARS‐CoV‐2 Nsp3 protein and human Parp14/Parp9 for the consensus amino acid sequence are shown in Table 3. This data showed that the amino acids of Nsp3, ORF1ab, and ORF1a from SARS‐CoV‐2 and from human PARP14 and PARP9 present an area of antigen–antibody complex formation. Amino acids of human PARP14 and PARP9 highlighted in gray even if different than the predicted Nsp3 viral epitope, present good antigenic and hydrophobic properties to form an antigen–antibody complex. This method can determine which portion of a protein will end up in the interior or on the outer side of said protein as a characteristic of most optimal antigenic epitopes is to be flexible, hydrophilic, and lie on the surface of the protein.

**Table 3:** Hydrophobicity and antigenicity prediction by the Kyte–Doolittle and Jameson–Wolf algorithms

SARS‐CoV‐2 Nsp3				Human Parp9				Human Parp14			
Residue	Position	Hydrophobyticy	Antigenic	Residue	Position	Hydrophobyticy	Antigenic	Residue	Position	Hydrophobyticy	Antigenic
Pro	236	−0.31	0.45								
Thr	237	−0.36	−0.45								
Val	238	−0.99	−0.3					Val	818	−1.53	−0.6
Val	239	−0.72	−0.6	Val	113	−1.42	−0.3	Val	819	−1.02	−0.6
Val	240	−0.77	−0.6	Val	114	−0.83	−0.3	Val	820	−0.81	−0.3
Asn	241	−1.41	−0.6	Asn	115	0.02	−0.6	Asn	821	0.04	−0.2
Ala	242	−1.34	−0.6	Ala	116	0.02	−0.3	Ala	822	0.04	1
Ala	243	−1.3	−0.6	Ala	117	−0.2	0.75	Ser	823	0.09	1.3
Asn	244	−0.4	−0.6	Asn	118	−0.16	0.75	Asn	824	0.99	1.3
Val	245	0.42	−0.6	Glu	119	0.67	0.45	Glu	825	1.81	0.9
Tyr	246	0.08	−0.6	Asp	120	0.32	0.45	Asp	826	1.57	0.9
Leu	247	0.32	−0.6	Leu	121	0.57	0.45	Leu	827	1.81	0.9
Lys	248	0.57	−0.6	Leu	122	0.81	0.3	Lys	828	1.77	0.9
His	249	−0.29	0.25	His	123	0	0.25	His	829	0.96	0.7
Gly	250	−0.02	0.65	Gly	124	−0.59	−0.05				
Gly	251	−0.12	0.85	Gly	125	−1.4	−0.05				
Gly	252	0.1	0.25	Gly	126	−1.18	−0.05				
Val	253	−0.76	−0.3	Leu	127	−1.18	−0.6				
Ala	254	−0.72	−0.6	Ala	128	−2	−0.6				
Gly	255	−0.33	−0.6								
Ala	256	−0.58	−0.3								

### 3.4 Prediction of the 3D structures of the predicted epitope and MHC I HLA‐A 0201 allele and MHC II HLA‐DR52c allele molecular docking

**Figure 3:**
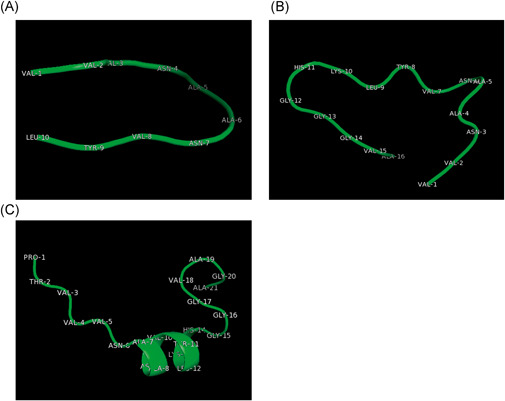
Epitope 3D model for VVVNAANVYLKH (A) against Parp14, VVNAANVYLKHGGGVA against Parp9 (B), and PTVVVNAANVYLKHGGGVAGA (C) against both Parp14/Parp9, was representative of the best clusters

**Figure 4:**
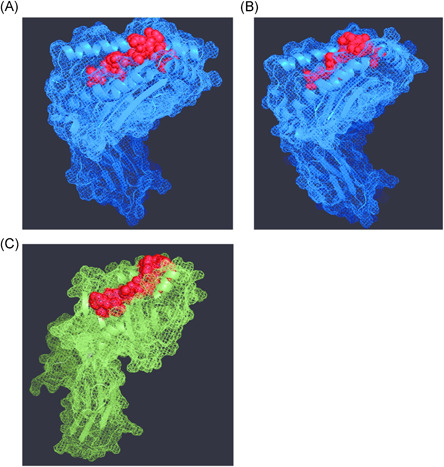
Molecular docking of the peptide: (A) Parp14 VVVNAANVYLKH epitope with MHC Class I HLA‐A*02:01 allele had binding free of −17.59 kcal/mol. (B) Parp9 VVNAANVYLKHGGGVA epitope with MHC Class I HLA‐A*02:01 allele had binding free of −5.97 kcal/mol. (C) Parp14/Parp9 epitope PTVVVNAANVYLKHGGGVAGA with MHC Class II HLA‐DR52c allele had binding free of −37.17 kcal/mol

Figure 3 shows the 3D epitope prediction. The sOPEP energy for the predicted epitope VVVNAANVYLKH was −8.54667 kcal/mol; with protein‐peptide docking showing an interface between the MHC Class I receptor HLA‐A*02:01 allele and peptide‐binding energy of −17.59 kcal/mol (Figure 4A). The sOPEP energy for the predicted epitope VVNAANVYLKHGGGVA was −9.76096 kcal/mol; with protein‐peptide docking showing an interface between the MHC Class I receptor HLA‐A*02:01 allele and peptide binding energy of −5.97 kcal/mol (Figure 4B). The sOPEP energy for the predicted epitope PTVVVNAANVYLKHGGGVAGA was −18.5287 kcal/mol; with protein‐peptide docking showing an interface between the MHC Class II receptor HLA‐DR52c allele and peptide‐binding energy of −37.17 kcal/mol (Figure 4C). These results suggest that the predicted epitopes may have a high binding affinity with MHC Class I and MHC Class II and are capable to direct immune response.

## 4 DISCUSSION

SARS‐CoV‐2 is a new and serious infectious disease that affects the entire world, threatening the health and life of the human population.^28^ The infection produced by the virus results in a strong immune response that releases large amounts of cytokines and chemokines, a phenomenon known as cytokine storm, exhibiting systemic hyper‐inflammation, leading to a high incidence of immune disorders, organ failure, and mortality.^11^, ^29^, ^30^ This systemic hyper‐inflammatory condition is known as MAS that can be associated with SARS‐CoV‐2 pneumonia and its exacerbation.^11^, ^12^

MAS can occur in severe infections caused by a wide variety of bacterial, fungal, protozoal, rickettsial, and viral pathogens,^31^, ^32^, ^33^ MAS is also found in patients with severe sepsis with a high risk of mortality.^34^ In patients with severe SARS‐CoV‐2, the MAS profile related to inflammatory macrophage M1^35^ may be explained, in part, by the systemic cytokine profiles observed in these cases, with increased production of IL‐6, IL‐7, and TNF‐α and inflammatory chemokines such as CCL2, CCl3, and CXC10.^36^ However, the amounts of IL‐1β serum levels were not increased in SARS‐CoV‐2 patients, which could indicate the absence of the inflammasome activation.^36^ Other characteristics found in a typical MAS profile, such as hypercytokinemia, elevated amounts of serum ferritin, CRP, and d‐dimer levels are the same as those found in patients with SARS‐Cov‐2 pneumonia.^36^

The polarization of macrophages is crucial to whether their function is pro‐inflammatory (M1) or anti‐inflammatory (M2). The M1 phenotype is activated by IFN‐γ, TNF‐α, IL‐1, IL‐6, IL‐12, IL‐23, and LPS to produce an inflammatory response.^37^ On the other hand, cytokines IL‐4 and IL‐13 produce stimulation and differentiation of the M2 phenotype that promotes resolution of inflammation and wound healing.^37^ PARP14 contributes to an IL‐4‐induced gene expression, as a co‐activator, through interaction with cytokine‐induced signal transducers and activators of transcription 6 (STAT6).^17^, ^38^ PARP14 is important for the differentiation of naïve T helper (Th) cells to a Th2 phenotype that produces IL‐4, IL‐5, and IL‐13, and is as well responsible for the polarization of the macrophages towards the M2 macrophages phenotype.^17^ Thus, PARP14 deficiency accelerates the activation of M1 macrophages leading to an inflammatory state.^17^ On the other hand, PARP9 is an important molecule that provides protection against lethal viral infections through interferon responses,^39^ and as PARP14, also participates in the polarization of macrophages towards the M2 phenotype.^17^

This in silico study showed that the SARS‐CoV‐2 Nsp3 protein, is critical for the virus replication in cells,^40^ is encoded by ORF1a^41^ and/or ORF1ab^42^ and has similarity to human PARP14 and PARP9. Thus, PARP14 could be depressed by a phenomenon of molecular mimicry causing the polarization of the M1 macrophage phenotype triggering the SARS‐CoV‐2 related cytokine storm by a hyper‐inflammatory state as well as by the MAS. In this manner, this study demonstrated that PARP9 could be depressed by the same phenomenon causing anti‐inflammatory macrophage depletion and poor interferon signaling leading to weak host defense against viral infections. The hypothesis of this study is as follows: when SARS‐CoV‐2 enters the body, macrophages and infected cells activate an early immune response through TLRs (TLR4, TLR3, and TLR7) triggering the expression of pro‐inflammatory cytokines and Type I/II interferon genes.^43^ B‐1 cells, belonging to innate immunity, through MHC Class I, can recognize viral epitopes and produce natural IgM against SARS‐CoV‐19,^44^ furthermore, the study of the activation of immunity from MHC Class I allowed us to observe the possible aggressiveness of SARS‐CoV‐2 by mounting a CD8^+^ immune response.^45^ These CD8^+^ cells under unknown conditions may act as Tc‐APCs that can activate an antiviral immune response by presenting viral peptides to other specialized cells.^46^ On another hand, as the cells mainly present as macrophages, the viral epitope via MHC Class II may be present to enhance the production of selective antibodies against PARP14/PARP9 causing an alteration in the immune regulation, allowing the polarization and activation of the M1 macrophage phenotype leading to the hyper‐inflammatory state related to SARS‐CoV‐2. Inflammatory macrophages mainly release IL‐6, which is responsible for the SARS‐CoV‐2 related macrophage activation syndrome, however, the presence of this cytokine produces the decrease of NK and CD8^+^ T cells,^28^, ^47^ a condition found in patients with severe SARS‐CoV‐2 infections.^28^, ^48^, ^49^ This scenario produces in the SARS‐CoV‐2 patient a state of constant hyper‐inflammation, without macrophage immune regulation. The combination of the decrease of PARP14/PARP9 responsible for the polarization of anti‐inflammatory macrophages (M2) and a weak host's viral response due to the decrease of NK and CD8^+^ cells will lead to a fatal outcome for the patient.

The results of this study are in agreement with previous studies denoting the presence of molecular mimicry in SARS‐CoV‐2 between PARP14/PARP9 and the viral proteome^10^, ^18^ and its importance in the SARS‐CoV‐2 infection.^50^

## 5 CONCLUSION

Within the limits of this study, it can be assumed that, in patients with severe SARS‐CoV‐2 infections, a molecular mimicry phenomenon of human PARP14 and PARP9 may be present leading to a hyperinflammatory state due to the macrophage activation syndrome known as cytokine storm‐related to SARS‐CoV‐2.

## CONFLICT OF INTERESTS

The authors declare that there are no conflict of interests.
